# Efficacy of four anti-vascular endothelial growth factor agents and laser treatment for retinopathy of prematurity: A network meta-analysis

**DOI:** 10.17305/bb.2023.9829

**Published:** 2024-08-01

**Authors:** Yufei Xu, Guohua Deng, Jun Zhang, Jie Zhu, Zhinan Liu, Fan Xu, Dong Zhou

**Affiliations:** 1 Basic Medical Science Academy, Air Force Medical University, Xi’an, Shaanxi Province, China; 2 Department of Ophthalmology, Changzhou Third People’s Hospital, Changzhou Medical Center, Nanjing Medical University, Changzhou, Jiangsu Province, China; 3 Department of Disease Control, The PLA Joint Logistics Support Force of No. 987 Hospital, Baoji, Shaanxi Province, China

**Keywords:** Anti-vascular endothelial growth factor (anti-VEGF), aflibercept, bevacizumab, conbercept, ranibizumab, laser therapy, retinopathy of prematurity (ROP), network meta-analysis (NME)

## Abstract

This study undertakes a comprehensive comparison of five different interventions for the treatment of type-1 retinopathy of prematurity (ROP) and aggressive posterior ROP (APROP), offering insights into their relative efficacies and contributing to better clinical decision making. The aim of this study was to compare the efficacy of intravitreal aflibercept (IVA), intravitreal bevacizumab (IVB), intravitreal conbercept (IVC), intravitreal ranibizumab (IVR), and laser therapy in treating these conditions. We conducted a search for relevant randomized controlled trials (RCTs) in databases, namely, PubMed, Embase, Cochrane Library, Web of Science, and Ovid, focusing on these five treatment modalities for ROP. The quality of the included studies was evaluated using the Cochrane Risk of Bias Assessment Tool, and data analysis was performed using STATA software. The results from our network meta-analysis (NMA) indicated that IVA significantly prolonged the interval between initial treatment and relapse in patients, with a surface under the cumulative ranking curve (SUCRA) value of 99.1%. Additionally, patients in the IVB group had a significantly higher spherical equivalent refraction (SER) after surgery, with a SUCRA value of 84.4%. Furthermore, IVR was the most effective in reducing the duration of peripheral retinal vascularization, with a SUCRA value of 95.6%. However, no statistically significant differences were found in relapse rates among the five treatment options. Our analysis concludes that intravitreal injections of anti-vascular endothelial growth factor (anti-VEGF) drug monotherapy generally offer better outcomes than laser treatment for ROP. Nonetheless, additional RCTs are necessary to further evaluate the safety of anti-VEGF agents.

## Introduction

Retinopathy of prematurity (ROP) is a proliferative retinal disease that can lead to severe ocular sequelae, including blindness. It is currently a significant public health issue in India and other low- and middle-income countries [1]. The disease is caused by retinal vascular dysplasia [2], and common treatments include laser ablation, which inhibits angiogenesis and therefore reduces the risk of retinal detachment [3]. While laser therapy was previously the standard treatment for ROP, the use of anti-vascular endothelial growth factor (anti-VEGF) drugs is increasingly popular in clinical settings [4], since the VEGF plays a key role in angiogenesis. These drugs, such as the commonly used ranibizumab and bevacizumab, are crucial in angiogenesis inhibition, and numerous studies have compared them with laser therapy [5–7]. However, there is a relative lack of research on several other anti-VEGF agents, as well as on comparisons between the two monotherapies [8]. In this context, a network meta-analysis (NMA) can be a valuable tool in comparing the efficacy of multiple anti-VEGF agents in treating premature infants with ROP.

In this study, we employed an NMA to compare various treatment modalities for ROP, specifically laser therapy, intravitreal ranibizumab (IVR), intravitreal aflibercept (IVA), intravitreal bevacizumab (IVB), and intravitreal conbercept (IVC). Our objective was to evaluate the impact of these treatments on the recovery outcomes of ROP patients, thereby providing patients and clinicians with a better understanding of their effects. The comparison focused on several key outcomes, including the recurrence interval, spherical equivalent refraction (SER), the recurrence prevalence, and the duration of peripheral retinal vascularization.

## Materials and methods

### Search strategy

A comprehensive literature search was conducted across multiple databases, including PubMed, EMBASE, Cochrane, Web of Science, and Ovid, covering the period from 1980 to September 2022. The search strategy was constructed using the PICOS framework: (P) Population: individuals diagnosed with ROP; (I) Intervention: treatments including laser therapy, IVR, IVA, IVB, and IVC; (C) Comparator: each treatment group served as a control for the others; (O) Outcomes: various indicators to assess the treatment effectiveness for ROP; (S) Study type: randomized controlled trials (RCTs). Using PubMed as an example, the detailed search strategy is shown in Table 1.

**Table 1:** Detailed search strategy in PubMed database

**Search**	**Query**
#1	((((((Retinopathy of Prematurity [MeSH Major Topic]) OR (Prematurity Retinopathies [MeSH Terms])) OR (Prematurity Retinopathy [MeSH Terms])) OR (Retrolental Fibroplasia [MeSH Terms])) OR (Fibroplasia, Retrolental [MeSH Terms])) OR (Fibroplasias, Retrolental [MeSH Terms])) OR (Retrolental Fibroplasias [MeSH Terms])
#2	(((((((Bevacizumab [MeSH Major Topic]) OR (technetium 99m tricarbonyl bevacizumab [Supplementary Concept])) OR (89Zr-bevacizumab [Supplementary Concept])) OR (byooviz [Supplementary Concept])) OR (Ranibizumab [MeSH Major Topic])) OR (aflibercept [Supplementary Concept])) OR (KH902 fusion protein [Supplementary Concept])) OR (conbercept [MeSH Terms]) OR (anti-VEGF drugs [Title/Abstract])
#3	(#1) AND (#2)
#4	((((SER) OR (SEQ)) OR (recurrence interval)) OR (recurrence prevalence)) OR (vascularization of peripheral retina)
#5	(#3) AND (#4)

MeSH: Medical subject headings; 89Zr: Zirconium-89; VEGF: Vascular endothelial growth factor; SER: Spherical equivalent refraction; SEQ: Spherical equivalent.

### Inclusion criteria

The inclusion criteria were as follows: (1) The study participants were patients diagnosed with type 1 ROP or aggressive posterior ROP (APROP). (2) Comparative studies comparing any two of the following five treatment methods for ROP, namely, IVR, IVA, IVB, IVC, and laser therapy, were considered. (3) All relevant and available RCTs were incorporated. (4) Outcomes were defined as follows: the recurrence interval, SER, the recurrence prevalence, and the duration of peripheral retinal vascularization.

### Exclusion criteria

The exclusion criteria were as follows: (1) Literature not relevant to the specified interventions or outcomes, or not meeting the established inclusion criteria. (2) Reports that are duplicates or studies lacking original data. (3) Studies involving patients not diagnosed with either type 1 ROP or APROP. (4) Studies derived from quasi-RCTs, case reports, animal studies, editorials, letters to the editor, conference abstracts, or reviews.

### Data extraction and quality assessment

The following pre-selected data were extracted from each study: first author, year of publication, gestational age (GA), type of ROP, sample size, details of intervention and control groups, the time interval between initial treatment and retreatment (i.e., recurrence interval), means and standard deviations (SD) of SER, recurrence prevalence, and duration of peripheral retinal vascularization.

The risk of bias (ROB) in the RCTs was assessed using the Review Manager Version 5.4.1 tool. The following seven parameters were considered: (1) randomized sequence generation; (2) allocation concealment; (3) blinding of participants and personnel; (4) blinding of outcome assessment; (5) completeness of outcome data; (6) selective reporting; and (7) potential for other biases. Each trial was categorized into one of the three ROB levels: high ROB, unclear ROB, and low ROB [9].

### Statistical analysis

In the intervention studies, all variables were continuous, except for the recurrence rate, which could be considered as a dichotomous variable. For the analysis of dichotomous variables, the odds ratio (OR) was employed, while the weighted mean difference (WMD) was used for continuous variables. Continuous data were presented as mean and range values, with SDs calculated using a statistical algorithm [10]. The meta-analysis was entirely conducted using STATA SE Version 15.0. Given the observed heterogeneity among the studies, a random effects model was employed for this meta-analysis [11].

Markov chain Monte Carlo simulation chains were utilized for NMA aggregation within a Bayesian framework [12, 13]. This approach enables the quantification of consistency between direct and indirect comparisons through the node-splitting method. Significance was set at a *P* value of less than 0.05. A *P* value greater than 0.05, as calculated by the software, indicates passing the consistency test [14]. Mean differences (MD) were calculated along with a 95% confidence interval (95% CI).

Network diagrams were generated in STATA, representing the various intervention and control conditions. The size of each node and the thickness of connecting lines correspond to the number of patients and the quantity of the included relevant publications, respectively [15].

The effectiveness of each treatment was evaluated and ranked, with the best treatment determined using the surface under the cumulative ranking curve (SUCRA) metric. The metric illustrates the percentage of effectiveness for each treatment, taking into account all possible rankings and uncertainties in treatment efficacy. SUCRA values range from 0, indicating the least effective treatment with no uncertainty, to 1, denoting the most effective treatment with no uncertainty [16]. However, it is important to note that the differences in SUCRA values do not reflect whether the differences between treatments are clinically significant, and thus, these results should be interpreted with caution [17].

**Figure 1.**
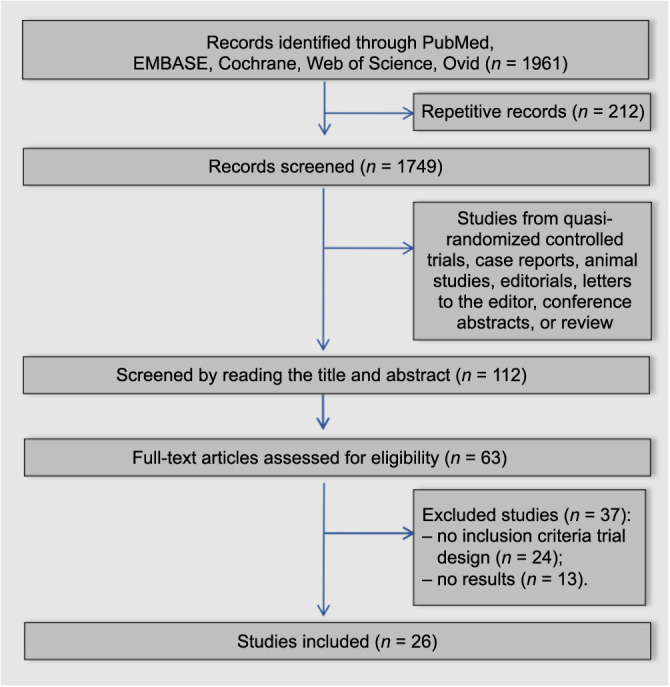
Flow diagram presenting the selection strategy.

**Figure 2.**
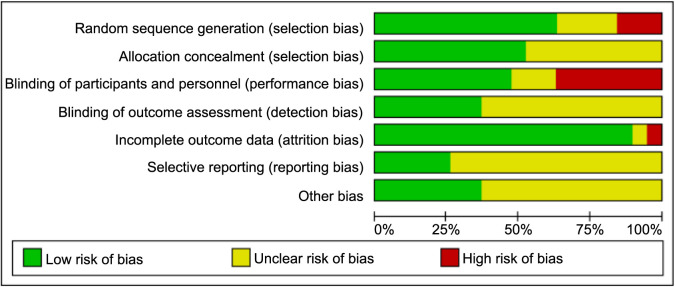
**Graph illustrating the risk of bias within the 26 included RCTs, across the different biases.** RCTs: Randomized controlled trials.

**Figure 3.**
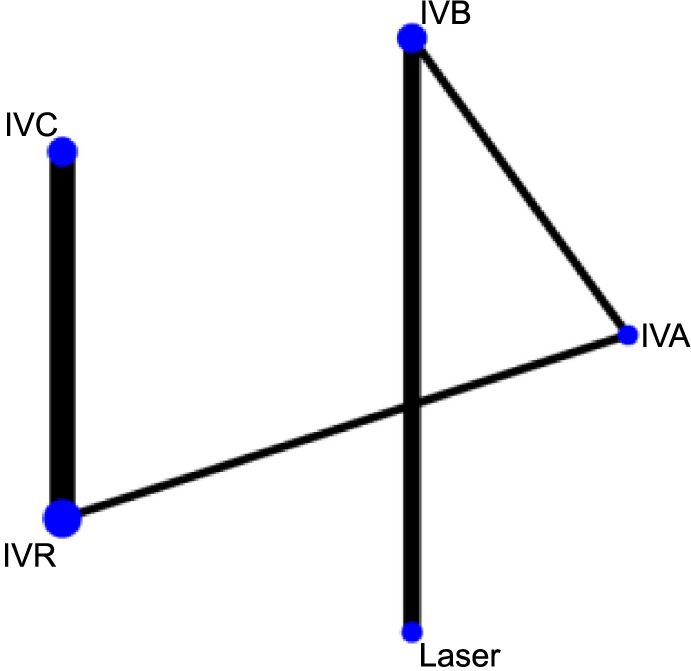
**Network graph generated for the recurrence interval of each intervention.** Each node in the network graph corresponds to a specific intervention within the NMA, with the node size representing the intervention’s relative weight. The thickness of connecting lines corresponds to the quantity of the included relevant publications. NMA: Network meta-analysis; IVC: Intravitreal conbercept; IVR: Intravitreal ranibizumab; IVA: Intravitreal aflibercept; IVB: Intravitreal bevacizumab.

**Figure 4.**
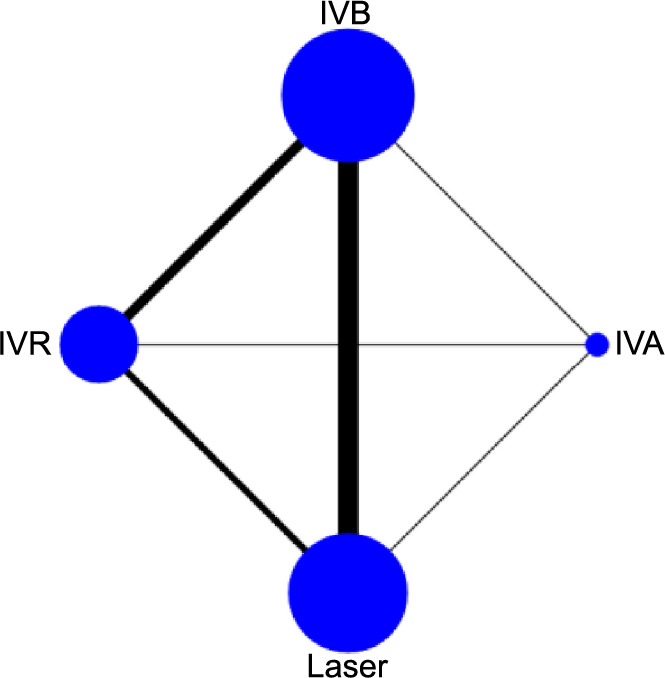
**Network graph generated for the SER of each intervention.** Each node in the network graph corresponds to a specific intervention within the NMA, with the node size representing the intervention’s relative weight. The thickness of connecting lines corresponds to the quantity of the included relevant publications. SER: Spherical equivalent refraction; NMA: Network meta-analysis; IVB: Intravitreal bevacizumab; IVA: Intravitreal aflibercept; IVR: Intravitreal ranibizumab.

**Figure 5.**
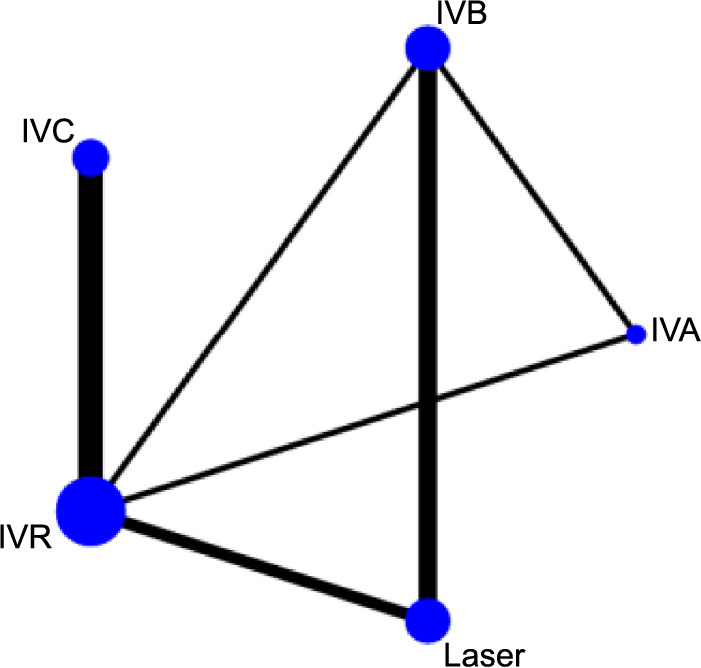
**Network graph generated for the recurrence prevalence of each intervention.** Each node in the network graph corresponds to a specific intervention within the NMA, with the node size representing the intervention’s relative weight. The thickness of connecting lines corresponds to the quantity of the included relevant publications. NMA: Network meta-analysis; IVA: Intravitreal aflibercept; IVB: Intravitreal bevacizumab; IVR: Intravitreal ranibizumab; IVC: Intravitreal conbercept.

**Figure 6.**
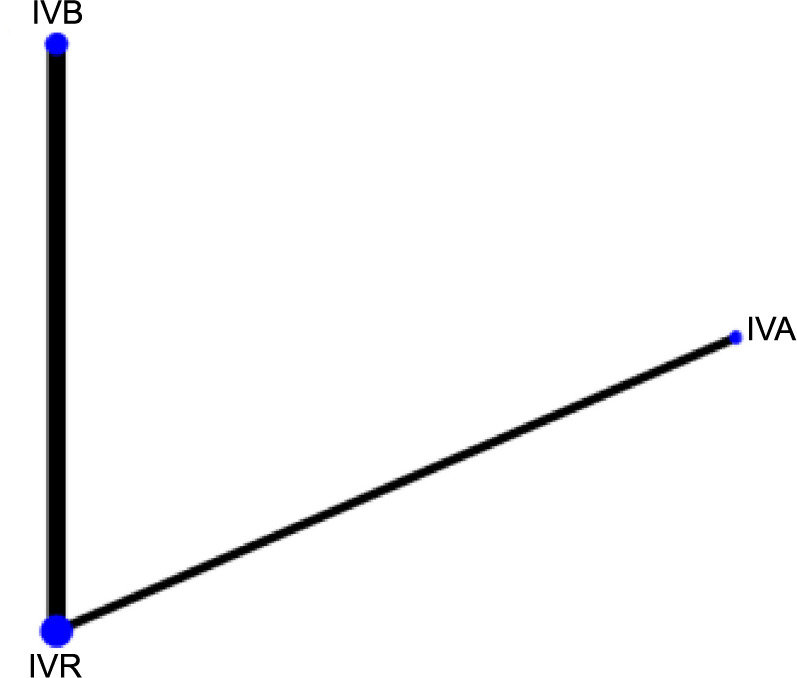
**Network graph generated for the duration of peripheral retinal vascularization of each intervention.** Each node in the network graph corresponds to a specific intervention within the NMA, with the node size representing the intervention’s relative weight. The thickness of connecting lines corresponds to the quantity of the included relevant publications. NMA: Network meta-analysis; IVA: Intravitreal aflibercept; IVR: Intravitreal ranibizumab; IVB: Intravitreal bevacizumab.

To address potential biases from smaller studies, funnel plot analysis was employed to visually assess the presence of publication bias [10, 18].

## Results

### Literature selection and study characteristics

A total of 1961 articles relevant to the study were initially retrieved from five electronic databases using the predetermined search strategy. From these, 212 duplicates were removed. Subsequently, 1637 publications, which were belonging to one of the following study types, namely, quasi-RCTs, case reports, animal studies, editorials, letters to the editor, conference abstracts, and reviews, were excluded. Following a review of the titles and abstracts of the remaining 112 articles, an additional 49 documents were excluded. The full texts of the remaining 63 documents were then thoroughly examined, leading to the exclusion of 37 more documents due to incompleteness. Ultimately, 26 documents met all the inclusion criteria and were included in this study. The process of screening and selecting the literature is detailed in the flow diagram presented in Figure 1.

A total of 3584 eyes affected by ROP were treated across the 26 included studies. The treatment distribution was as follows: 978 eyes were treated with IVR, 96 with IVA, 1556 with IVB, 175 with IVC, and 779 with laser therapy [19–44]. Given that several studies distinguished between treatments for different ROP types [19, 21, 27, 39], data were ultimately aggregated for 30 distinct treatment pairs. The literature reporting primary outcome data for the various ROP treatments is summarized in Table S1.

### Study quality assessment

The quality assessment of the 26 included RCTs is presented in Figure 2. Of these, three studies were categorized as low-risk, 13 as high-risk, and ten as moderate-risk.

### Network meta-analysis

Figures 3–6 display the comprehensive NMA results, presented as network graphs, for various outcomes.

### Recurrence interval

Table 2 presents the results of consistency and inconsistency tests for indirect and direct comparisons across the studies. The *P* values for these tests were all above 0.05, suggesting that any inconsistencies among the studies were not statistically significant, thereby validating the use of a consistency model for analysis.

**Table 2:** Consistency test of recurrence interval

**Side**	**Direct**		**Indirect**		**Difference**		***P* > |*z*|**	**tau**
	**Coef.**	**Std. Err.**	**Coef.**	**Std. Err.**	**Coef.**	**Std. Err.**		
A vs B	−8.15	1.823489	0.000175	223.6188	−8.15017	223.6262	0.971	1.570431
A vs D	−6	1.580167	−2.49847	182.6026	−3.50153	182.6095	0.985	1.570515
B vs E	−9.26407	1.666017	7.035966	447.2542	−16.3	447.2539	0.971	1.570429
C vs D	−2.49847	1.10721	−9.50159	365.0447	7.00312	365.0472	0.985	1.570514

A: Intravitreal aflibercept; B: Intravitreal bevacizumab; C: Intravitreal conbercept; D: Intravitreal ranibizumab; E: Laser; Coef.: Coefficient; Std. Err.: Standard error.

**Figure 7.**
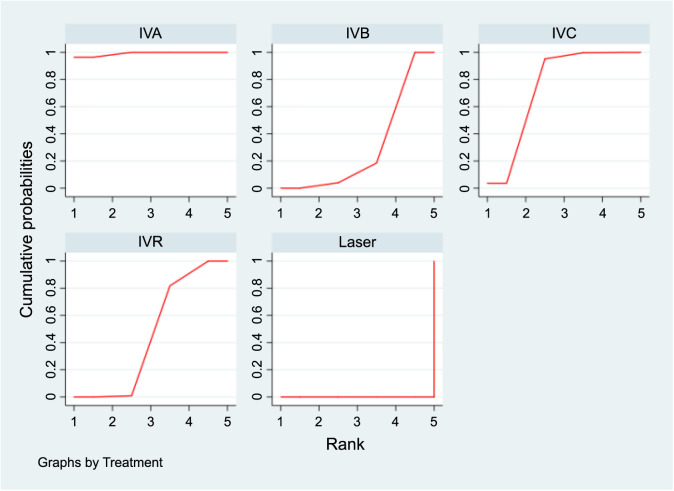
**SUCRA plots displaying the probability of each interventions’s effectiveness in extending the recurrence interval for ROP.** SUCRA: Surface under the cumulative ranking curve; ROP: Retinopathy of prematurity; IVA: Intravitreal aflibercept; IVB: Intravitreal bevacizumab; IVC: Intravitreal conbercept; IVR: Intravitreal ranibizumab.

In the SUCRA analysis, IVA emerged as the leading treatment option in terms of increasing the recurrence interval among the different treatments, with a SUCRA probability of 99.1%, as detailed in Figure 7. The NMA results showed that IVA (MD ═ 17.41, 95% CI 12.57–22.25), IVC (MD ═ 13.91, 95% CI 7.96–19.86), IVR (MD ═ 11.41, 95% CI 5.67–17.16), and IVB (MD ═ 9.26, 95% CI 6.00–12.53) all demonstrated superiority over laser therapy in terms of prolonging the recurrence interval. Specifically, IVA was found to be superior to the IVB group (MD ═ 8.15, 95% CI 4.58–11.72). Additionally, compared to the IVR group, both IVA (MD ═ 6.00, 95% CI 2.90–9.10) and IVC (MD ═ 2.50, 95% CI 0.33–4.67) showed better outcomes in extending the recurrence interval, as summarized in Table 3.

**Table 3:** League table comparing interventions in terms of recurrence interval for ROP

**IVA**	**IVC**	**IVR**	**IVB**	**Laser**
IVA	−3.50 (−7.28, 0.28)	−6.00 (−9.10, −2.90)	−8.15 (−11.72, −4.58)	−17.41 (−22.25, −12.57)
3.50 (−0.28, 7.28)	IVC	−2.50 (−4.67, −0.33)	−4.65 (−9.85, 0.55)	−13.91 (−19.86, −7.96)
6.00 (2.90, 9.10)	2.50 (0.33, 4.67)	IVR	−2.15 (−6.88, 2.58)	−11.41 (−17.16, −5.67)
8.15 (4.58, 11.72)	4.65 (−0.55, 9.85)	2.15 (−2.58, 6.88)	IVB	−9.26 (−12.53, −6.00)
17.41 (12.57, 22.25)	13.91 (7.96, 19.86)	11.41 (5.67, 17.16)	9.26 (6.00, 12.53)	Laser

ROP: Retinopathy of prematurity; IVC: Intravitreal conbercept; IVR: Intravitreal ranibizumab; IVB: Intravitreal bevacizumab; IVA: Intravitreal aflibercept.

**Figure 8.**
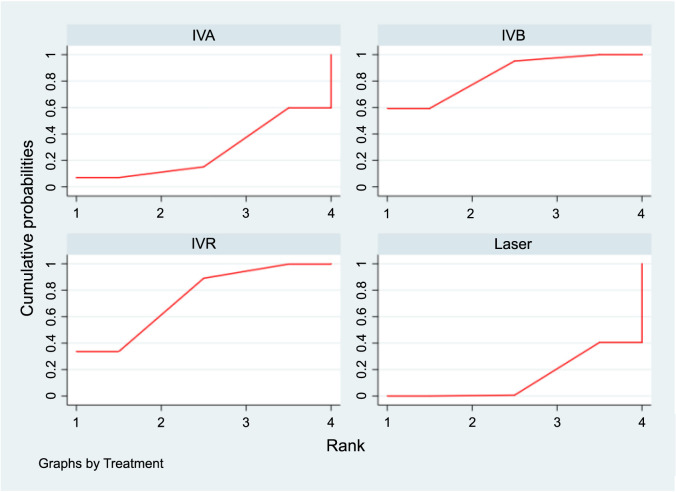
**SUCRA plots displaying the probability of each interventions’s effectiveness in improving SER for ROP.** SUCRA: Surface under the cumulative ranking curve; SER: Spherical equivalent refraction; ROP: Retinopathy of prematurity; IVA: Intravitreal aflibercept; IVB: Intravitreal bevacizumab; IVR: Intravitreal ranibizumab.

### Spherical equivalent refraction

Table 4 presents the results of consistency and inconsistency tests for indirect and direct comparisons across the studies. The *P* values for these tests were all above 0.05, suggesting that any inconsistencies among the studies were not statistically significant, thereby validating the use of a consistency model for analysis. In the SUCRA analysis, IVB emerged as the leading treatment option for achieving a higher SER, with a SUCRA probability of 84.8%, as illustrated in Figure 8. The NMA results showed that IVB (MD ═ 2.26, 95% CI 1.05–3.47) and IVR (MD ═ 1.97, 95% CI 0.39–3.55) demonstrated superiority over laser therapy in terms of improving SER, as detailed in Table 5.

**Table 4:** Consistency test of SER

**Side**	**Direct**		**Indirect**		**Difference**		***P* > |*z*|**	**tau**
	**Coef.**	**Std. Err.**	**Coef.**	**Std. Err.**	**Coef.**	**Std. Err.**		
A vs B	1.231139	2.225711	2.462118	2.013416	−1.23098	3.053236	0.687	2.066299
A vs C	1.121507	2.099399	2.118603	2.144911	−0.9971	3.009179	0.74	2.071488
A vs D	0.4	2.168979	−0.98125	1.953319	1.381254	2.918891	0.636	2.066141
B vs C	0.34925	0.852151	−2.31225	1.528728	2.661496	1.748717	0.128	1.937368
B vs D	−2.61744	0.649196	−0.0217	1.577848	−2.59574	1.705747	0.128	1.94004
C vs D	−0.8418	1.033442	−3.43224	1.185539	2.590441	1.571817	0.099	1.929221

A: Intravitreal aflibercept; B: Intravitreal bevacizumab; C: Intravitreal ranibizumab; D: Laser; SER: Spherical equivalent refraction; Coef.: Coefficient; Std. Err.: Standard error.

**Table 5:** League table comparing interventions in terms of SER for ROP

**IVB**	**IVR**	**IVA**	**Laser**
IVB	−0.29 (−1.79, 1.21)	−1.90 (−4.70, 0.90)	−2.26 (−3.47, −1.05)
0.29 (−1.21, 1.79)	IVR	−1.61 (−4.45, 1.24)	−1.97 (−3.55, −0.39)
1.90 (−0.90, 4.70)	1.61 (−1.24, 4.45)	IVA	−0.36 (−3.13, 2.41)
2.26 (1.05, 3.47)	1.97 (0.39, 3.55)	0.36 (−2.41, 3.13)	Laser

SER: Spherical equivalent refraction; ROP: Retinopathy of prematurity; IVB: Intravitreal bevacizumab; IVR: Intravitreal ranibizumab; IVA: Intravitreal aflibercept.

### Recurrence prevalence

Table 6 presents the results of consistency and inconsistency tests for indirect and direct comparisons across the studies. The *P* values for these tests were all above 0.05, suggesting that any inconsistencies among the studies were not statistically significant, thereby validating the use of a consistency model for analysis.

**Table 6:** Consistency test of recurrence prevalence

**Side**	**Direct**		**Indirect**		**Difference**		*P* > |*z*|	**tau**
	**Coef.**	**Std. Err.**	**Coef.**	**Std. Err.**	**Coef.**	**Std. Err.**		
A vs B	−6.31385	5.234515	0.710127	4.364904	−7.02398	6.81561	0.303	7.55E^−09^
A vs D	1.53609	3.050549	−5.47086	6.087188	7.006949	6.808796	0.303	5.52E^−09^
B vs D	0.019697	4.905611	3.37595	3.368964	−3.35625	5.951102	0.573	7.64E^−09^
B vs E	1.737444	3.259879	3.484009	4.51025	−1.74657	5.565014	0.754	1.33E^−08^
C vs D	−0.36457	1.449255	0.624001	316.1691	−0.98857	316.1791	0.998	5.78E^−09^
D vs E	0.363807	2.410035	−1.38149	5.01565	1.745293	5.564529	0.754	8.25E^−09^

A: Intravitreal aflibercept; B: Intravitreal bevacizumab; C: Intravitreal conbercept; D: Intravitreal ranibizumab; E: Laser; Coef.: Coefficient; Std. Err.: Standard error.

**Figure 9.**
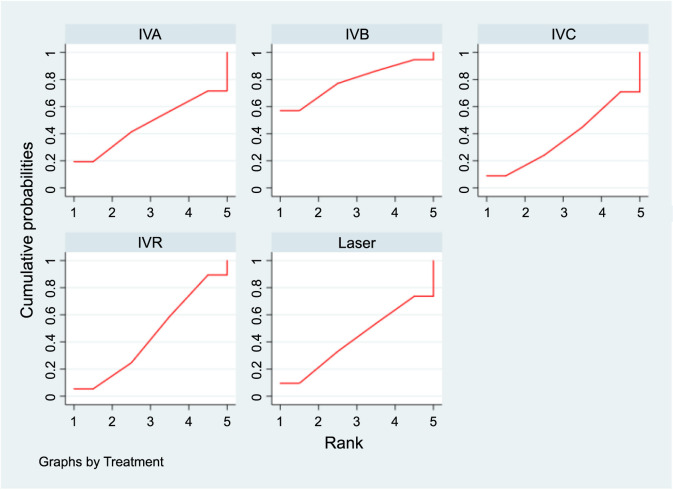
**SUCRA plots displaying the probability of each interventions’s effectiveness in reducing recurrence prevalence.** SUCRA: Surface under the cumulative ranking curve; IVA: Intravitreal aflibercept; IVB: Intravitreal bevacizumab; IVC: Intravitreal conbercept; IVR: Intravitreal ranibizumab.

Figure 9 presents the SUCRA results for the five interventions under study. Based on the findings in Table 7, no significant differences were observed in the impact of the various treatments on the recurrence rate of ROP.

**Table 7:** League table comparing interventions in terms of recurrence rate of ROP

**IVB**	**IVA**	**IVR**	**Laser**	**IVC**
IVB	2.17 (−4.40, 8.74)	2.30 (−3.14, 7.74)	2.34 (−2.84, 7.52)	2.66 (−3.47, 8.80)
−2.17 (−8.74, 4.40)	IVA	0.13 (−5.22, 5.47)	0.17 (−6.27, 6.60)	0.49 (−5.56, 6.55)
−2.30 (−7.74, 3.14)	−0.13 (−5.47, 5.22)	IVR	0.04 (−4.22, 4.29)	0.36 (−2.48, 3.20)
−2.34 (−7.52, 2.84)	−0.17 (−6.60, 6.27)	−0.04 (−4.29, 4.22)	Laser	0.33 (−4.79, 5.45)
−2.66 (−8.80, 3.47)	−0.49 (−6.55, 5.56)	−0.36 (−3.20, 2.48)	−0.33 (−5.45, 4.79)	IVC

ROP: Retinopathy of prematurity; IVB: Intravitreal bevacizumab; IVA: Intravitreal aflibercept; IVR: Intravitreal ranibizumab; IVC: Intravitreal conbercept.

**Figure 10.**
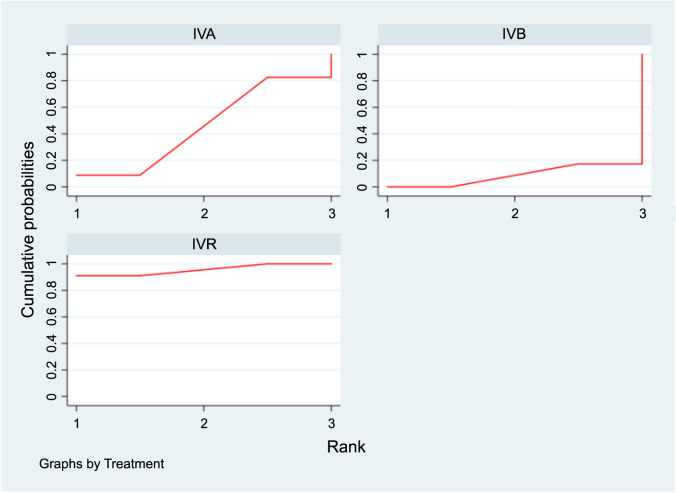
**SUCRA plots displaying the probability of each interventions’s effectiveness in reducing the duration of peripheral retinal vascularization.** SUCRA: Surface under the cumulative ranking curve; IVA: Intravitreal aflibercept; IVB: Intravitreal bevacizumab; IVR: Intravitreal ranibizumab.

### Duration of peripheral retinal vascularization

Table 8 presents the results of consistency and inconsistency tests for indirect and direct comparisons across the studies. The *P* values for these tests were all above 0.05, suggesting that any inconsistencies among the studies were not statistically significant, thereby validating the use of a consistency model for analysis.

**Table 8:** Consistency test of the duration of peripheral retinal vascularization

**Side**	**Direct**		**Indirect**		**Difference**		***P* > |*z*|**	**tau**
	**Coef.**	**Std. Err.**	**Coef.**	**Std. Err.**	**Coef.**	**Std. Err.**		
A vs C	−8.68	6.374581	−6.30327	223.6554	7.623271	223.7455	0.973	6.175128
B vs C	−16.30327	4.703271	−1.05673	447.4654	−15.24654	447.4936	0.973	6.175129

A: Intravitreal aflibercept; B: Intravitreal bevacizumab; C: Intravitreal ranibizumab; Coef.: Coefficient; Std. Err.: Standard error.

In the SUCRA analysis, IVR emerged as the leading treatment option in reducing the duration of peripheral retinal vascularization, with a SUCRA probability of 95.6%, as illustrated in Figure 10. The NMA results showed that IVR (MD ═ −16.30, 95% CI −25.51 to −7.09) demonstrated superiority over the IVB group in terms of decreasing the duration of peripheral retinal vascularization, as detailed in Table 9.

**Table 9:** League table comparing interventions in terms of the duration of peripheral retinal vascularization in ROP

**IVR**	**IVA**	**IVB**
IVR	8.69 (−3.79, 21.17)	16.30 (7.09, 25.51)
−8.69 (−21.17, 3.79)	IVA	7.61 (−7.89, 23.12)
−16.30 (−25.51, −7.09)	−7.61 (−23.12, 7.89)	IVB

ROP: Retinopathy of prematurity; IVR: Intravitreal ranibizumab; IVA: Intravitreal aflibercept; IVB: Intravitreal bevacizumab.

### Publication bias test

**Figure 11.**
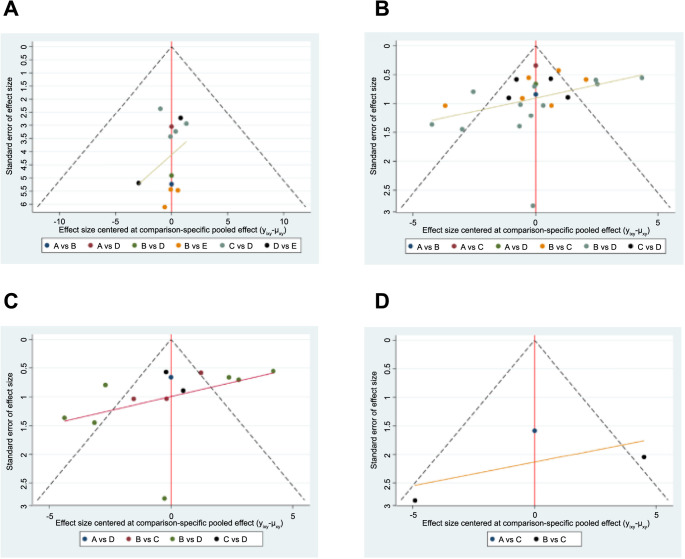
**Funnel plots assessing publication bias for various outcomes.** (A) Recurrence interval; (B) Spherical equivalent refraction; (C) Recurrence rate; (D) Duration of peripheral retinal vascularization. A: Intravitreal aflibercept; B: Intravitreal bevacizumab; C: Intravitreal conbercept; D: Intravitreal ranibizumab; E: Laser.

Funnel plots were constructed to assess publication bias for all outcomes, as shown in Figure 11, and no significant publication bias was found.

## Discussion

This NMA encompassed 26 studies involving 3584 eyes with ROP. It compared the efficacy of four anti-VEGF monotherapies and laser treatments for ROP across four outcomes. The results showed that intravitreal anti-VEGF injections resulted in a longer recurrence interval compared to laser therapy. Specifically, IVA treatment resulted in a longer recurrence interval compared to IVC, and subsequently longer than both IVR and IVB. However, no significant difference in recurrence interval was observed between IVR and IVB. Regarding the higher SER, IVB emerged as the most effective treatment, significantly outperforming laser therapy. This suggests that IVB can reduce postoperative myopia. The IVB approach also led to the shortest duration of peripheral retinal vascularization after operation, indicating quicker healing. Overall, there were no significant differences in the relapse rate outcome between the five treatments, a finding which is inconsistent with previous studies [8]. This inconsistency could be attributed to the inclusion of more databases covering a broader range of RCTs, while excluding retrospective studies. Additionally, the increasing clinical use of anti-VEGF drugs over time has resulted in the availability of more comprehensive study data.

This NMA possesses notable strengths as well as limitations. A key strength lies in its larger sample size and more comprehensive data sources compared to previous studies, which primarily focused on bevacizumab, ranibizumab, and laser [7, 45, 46], Additionally, this study incorporated two other clinically relevant agents, aflibercept and conbercept [47], enabling a simultaneous comparison of five treatment methods and thus offering more comprehensive, evidence-based clinical insights. However, there are also some limitations. The less frequent clinical use of conbercept in the ROP treatment has resulted in a scarcity of available data for inclusion, thereby limiting the extent to which it can be compared across all outcome indicators. For instance, the absence of SER data in patients treated with IVC indirectly reduced the sample size and potentially increased the ROB. The limited availability of head-to-head direct comparative evidence for some interventions highlights the continued need for further expansion of relevant research in this area. Another limitation is the study’s lack of differentiation between various doses of the same drug, overlooking the potential effect of dosage on treatment efficacy and possibly introducing bias into the analysis. Finally, heterogeneity among the included studies was inevitable.

## Conclusion

In conclusion, our study holds clinical significance by demonstrating that anti-VEGF drugs, overall, have superior efficacy compared to laser treatment in managing ROP. However, given the varying benefits of different anti-VEGF drugs, the clinical choice of drug should be patient specific. Particularly, doctors should consider the safety of the drug, in addition to its effectiveness, in their decision making [40, 48].

## Supplemental data

**Table S1:** Characteristics of the studies included in the meta-analysis

**Author**	**Year**	**Population**	**GA (weeks)**	**Total/T/C**	**Intervention**	**Control**	**Outcome**
Cheng	2020	Zone I ROP and APROP	T: 27.3 ± 1.8 C: 28.0 ± 2.3	133/98/35	IVR	IVC	Recurrence interval, recurrence rate
Cheng	2020	Zone II ROP	T: 31.7 ± 1.9 C: 32.1 ± 1.7	492/382/110	IVR	IVC	Recurrence interval, recurrence rate
Sukgen	2019	Zone I/II ROP	T:28.35 ± 2.58 C: 28.3 ± 2.05	63/27/36	IVR	IVA	Recurrence interval, recurrence rate, vascularization of peripheral retina
Kabatas	2017	Type 1 ROP	–	108/24/12/72	IVB, IVR	Laser	SER, vascularization of peripheral retina
Geloneck	2014	Zone I ROP	–	87/52/35	IVB	Laser	Recurrence rate, SER
Geloneck	2014	Zone II ROP	–	124/58/66	IVB	Laser	Recurrence rate, SER
Tiryaki Demir	2021	Severe ROP	T: 29.4 ± 2.5 C: 29 ± 2.9	62/38/24	IVB	Laser	SER
Simmons	2021	Zone I/II ROP	T: 24.5 ± 1.3 C: 24.7 ± 1.2	48/22/26	IVB	Laser	SER
Zhao	2020	Zone II ROP	T: 37.4 ± 1.6 C: 36.9 ± 2.0	65/28/37	IVR	Laser	SER
Wu	2022	APROP and zone I or posterior zone II ROP	T: 28.27 ± 2.77 C: 27.50 ± 2.70	24/10/14	IVC	IVR	Recurrence interval, recurrence rate
Lin	2016	ROP	T: 26.15 ± 2.08 C: 26.50 ± 2.14	40/25/15	IVR	IVB	SER
Kimyon	2018	Type 1 ROP	T: 29.3 ± 2.6 C: 30.1 ± 2.4	62/36/26	IVB	IVR	SER, vascularization of peripheral retina
Ekinci	2020	Type 1 ROP or APROP	T: 34.2 ± 4.2 C: 37.6 ± 2.5	51/24/27	IVA	Laser	SER
Jin	2018	Zone I/II stage 2/3 ROP or APROP	T: 29.49 ± 1.37 C:28.35 ± 1.62	48/20/28	IVC	IVR	Recurrence rate
Zhang	2017	Zone II ROP	T: 28.96 ± 1.59 C: 28.27 ± 1.84	100/50/50	IVR	Laser	Recurrence rate
Riazi-Esfahani	2021	Type 1 ROP	T: 28.2 ± 2 C: 28.7 ± 2.3	889/865/24	IVB	IVA	Recurrence interval, recurrence rate, regression of plus disease
Isaac	2015	Type 1 ROP	T: 25.2 ± 1.4 C: 25.0 ± 1.1	45/23/22	IVB	Laser	SER
Stahl	2019	Zone I/II ROP	T: 25 (23–32) 26 (23–32) C: 26 (23–32)	225/74/77/74	IVR 0.2mg IVR 0.1mg	Laser	Recurrence rate
Mintz-Hittner	2011	Zone I ROP	T: 24.2 ± 1.3 C: 24.3 ± 1.6	67/33/34	IVB	Laser	Recurrence interval, recurrence rate
Mintz-Hittner	2011	Zone II posterior ROP	T: 24.5 ± 1.2 C: 24.5 ± 1.4	83/42/41	IVB	Laser	Recurrence interval, recurrence rate
Kuo	2015	ROP	T: 27.33 ± 2.94 C: 27.43 ± 2.93	53/27/26	IVB	Laser	SER
Chen	2020	Type 1 ROP	T: 26.46 ± 1.51 C: 25.50 ± 1.24	25/13/12	IVB	Laser	SER, recurrence rate
Lee	2018	Type 1 ROP	T: 26.6 ± 1.6 C: 26.6 ± 2.5	57/33/24	IVB	Laser	SER
Gunay	2017	Zone I ROP	–	264/107/44/113	IVB, IVR	Laser	SER, recurrence rate
Harder	2013	Zone I/II ROP	T: 25.2 ± 1.6 C: 25.3 ± 1.8	49/23/26	IVB	Laser	SER
Hwang	2015	Zone I ROP	T: 24.3 ± 1.0 C: 24.4 ± 0.0	21/16/5	IVB	Laser	SER, recurrence rate
Hwang	2015	Zone II ROP	T: 24.0 ± 1.0 C: 24.9 ± 1.3	33/6/27	IVB	Laser	SER, recurrence rate
Lu	2022	Zone II stage 3 ROP	T: 27.71 ± 1.81 C: 27.57 ± 1.95	55/28/27	IVR	Laser	SER
Sukgen	2022	Type 1 ROP or APROP	–	36/10/14/12	IVB, IVR	IVA	SER
Kang	2018	ROP	T: 26.9 ± 1.9 C: 28.1 ± 3.2	153/101/52	IVB	IVR	SER, recurrence rate

GA: Gestational age; T: Experimental group; C: Control group; ROP: Retinopathy of prematurity; APROP: Aggressive posterior retinopathy of prematurity; IVR: Intravitreal ranibizumab; IVC: Intravitreal conbercept; IVA: Intravitreal aflibercept; IVB: Intravitreal bevacizumab; SER: Spherical equivalent refractions.

## Data Availability

The datasets used in this study are available from the corresponding author, Dong Zhou.
